# Management of Pancreatic Calculi in Chronic Pancreatitis: A Review Article

**DOI:** 10.7759/cureus.35788

**Published:** 2023-03-05

**Authors:** Nikhil Kaushik, Venkatesh Dasari, Dhriti Jain

**Affiliations:** 1 Surgery, Jawaharlal Nehru Medical College, Datta Meghe Institute of Medical Sciences, Wardha, IND; 2 Orthopedics and Traumatology, Jawaharlal Nehru Medical College, Datta Meghe Institute of Medical Science, Wardha, IND; 3 Otolaryngology, Jawaharlal Nehru Medical College, Datta Meghe Institute of Medical Sciences, Wardha, IND

**Keywords:** extracorporeal shock wave lithotripsy, surgery, endoscopy, pancreatic calculi, chronic pancreatitis

## Abstract

Chronic pancreatitis is a slow, irreversible, and progressive inflammatory condition with abdominal pain, loss of parenchyma, fibrosis, and calculus formation. It also causes loss of exocrine and endocrine function. Gallstones and alcohol is the most frequent cause of chronic pancreatitis. It is also caused by other factors, including oxidative stress, fibrosis, and repeated incidence of acute pancreatitis. Chronic pancreatitis is followed by several sequelae, one of them being formation of calculi in the pancreas. The formation of calculi can occur in the main pancreatic duct, branches of the duct, and parenchyma. The cardinal sign of chronic pancreatitis is pain caused by obstruction of pancreatic ducts and its branches leading to ductal hypertension resulting in pain. The main aim of endotherapy includes pancreatic duct decompression. The management options vary based on the type and size of the calculus. The treatment of choice for small-sized pancreatic calculi is endoscopic retrograde cholangiopancreatography (ERCP) followed by sphincterotomy and extraction. The large-sized calculi need fragmentation before extraction, which is done by extracorporeal shock wave lithotripsy (ESWL). Surgery can be an option for patients having severe pancreatic calculi if endoscopic therapy fails. For diagnostic purposes, imaging plays a very important role. The treatment options remain complex if the radiological and laboratory findings overlap. Due to advancements in diagnostic imaging, treatment options have become precise and helpful. It can significantly lower the quality of life along with immediate and long-term problems that pose a serious risk to life. This review comprises the various management options available for removing calculi following chronic pancreatitis, including surgical, endoscopic, and medical therapy.

## Introduction and background

Chronic pancreatitis has various etiologies with irreversible morphological changes occurring in the pancreas as its characteristic feature [1]. It is one of the common gastrointestinal diseases [2]. Chronic pancreatitis is a persistent inflammatory condition that causes fibrotic remodeling of the tissue of the pancreas and loss of function of both its exocrine and endocrine part [3]. Amongst all the causes of the condition, alcohol is the most frequent one in the Western countries [4]. The other factors include gall stones, smoking, metabolic disturbances, and genetic and immunological defects [5]. Numerous mechanisms have been considered as the pathophysiology, like (i) the toxic metabolite effect on acinar cells directly (e.g., tobacco, alcohol), (ii) pancreatic stellate cells activated by an episode of acute pancreatitis with subsequent fibrosis, (iii) protein plug formation leading to obstruction and causing ductal dysfunction, (iv) free radicals causing oxidative stress, and (v) recurrent episodes of acute pancreatitis causing necrosis-fibrosis [6]. Chronic pancreatitis leads to the formation of pancreatic calculi. It leads to duct blockage, causing ductal hypertension and abdominal pain [7]. The stones occur in the pancreatic duct as the disease progresses and are found in 90% of patients [8]. The division of pancreatic calculi is done based on type, location, and numbers. They can be (1) radiolucent, radio-opaque, or mixed; (2) situated in the duct of pancreas, side branches or parenchyma of pancreas; (3) single or multiple; and (4) found in regions like the head, body, or tail [5]. It has been observed that pancreatic calculi in the nonalcoholic, idiopathic type of chronic pancreatitis are generally larger and denser than ones found in the population consuming alcohol [9]. Due to the pancreatic duct calculi, there can be pancreatic duct obstruction which results in upstream hypertension, a rise in pressure of parenchyma, and ischemia. The chief symptom in the patients of chronic pancreatitis is pain. One of the ways to decrease the symptom is by removing the pancreatic stones [10]. The endoscopic approach is less invasive than the surgical procedure but more successful in case of small-sized stones situated in the main pancreatic duct [11].

Methodology

**Figure 1:**
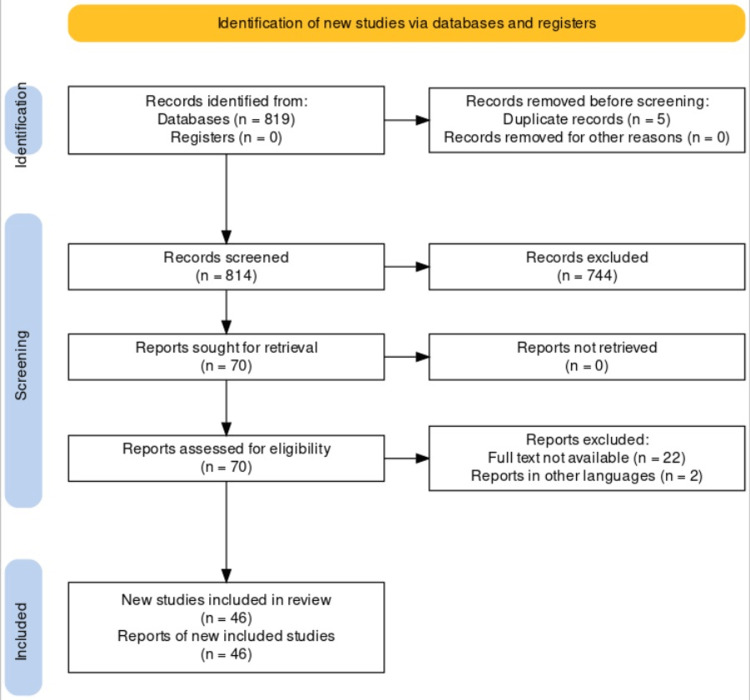
PRISMA flow diagram for pancreatic calculi and management.

A systematic search was undertaken through PubMed in November 2022 using keywords like “pancreatic” , “calculi” and “management” ((Pancreatic) AND (calculi)) AND (management). We also looked for the key references of relevant studies from their respective bibliographies. The search was updated in January 2023. One reviewer separately checked the titles, abstracts, and full texts of the retrieved papers to see if they met the inclusion criteria before including them. The steps for inclusion studies are depicted in Figure 1.

## Review

Pathophysiology

One of the major roles in forming pancreatic calculi is a pancreatic stone protein (PSP) [12]. There is a reduction in PSP due to numerous factors like gene expression and other related factors. Calcium carbonate becomes oversaturated in the pancreatic juice when PSP is reduced. There is then deposition of calcium carbonate over an inner nidus. All pancreatic calculi contain amorphous nidus comprising the center of the pancreatic stone. Nickel, chromium, and iron are among the substances found in the nidus. Calcium carbonate is deposited over the nidus through multiple layers and stages [13]. In cases of chronic pancreatitis associated with alcohol, there is decreased pancreatic stone protein secretion which results in the crystallization of calcium carbonate and its deposition leading to stone formation [14]. Pancreatic duct strictures result in pancreatic juice stagnation and promote the development of pancreatic stones. In patients with hyperparathyroidism, hypercalcemia may elevate the calcium levels in pancreatic secretion, hastening the development of stones in pancreas. The endoscopic treatment makes soft stones formed by intraductal protein precipitates comparatively easy to remove.

Diagnosis

The lab investigation includes tests for serum lipase which rises gradually and amylase which rises quickly in case of pancreatitis. It also includes tests for malabsorption that is exocrine insufficiency like fecal fat test and fecal elastase-1 test: in which <100 mg indicates malabsorption. The tests for endocrine insufficiency include blood and insulin levels. The most specific finding on plain abdomen images in patients of chronic pancreatitis is diffuse pancreatic calcifications. Focal calcifications are found in cases of peripancreatic vascular calcifications and cystic and islet cell tumors. Plain abdominal radiographs can be useful in showing calcification in the pancreas. Pancreatic calcification can be observed on plain abdominal X-rays in 30% of patients [15]. An obstructive hydrostatic effect will cause the duct of the pancreas to enlarge when pancreatic stones impede it, which might help identify the main duct stone. The dilated pancreatic duct and stones can be detected by ultrasonography, but in some cases, the pancreas is not visible due to overlying gas in the bowel [16]. Pancreatic calcification can be better identified by CT [17]. The information regarding size and location of the stones can be provided by endoscopic ultrasound [18]. Endoscopic retrograde cholangiopancreatography (ERCP) or magnetic resonance cholangiopancreatography provides more particular images of pancreatic stones and ducts [19].

Management

Pain is the most common feature seen in chronic pancreatitis patients, as stated above, and because of the presence of stones, pancreatic duct is obstructed which leads to an increase in upstream hypertension. This hypertension can be aggravated by a coexisting ductal structure [5]. Over the past few years, endoscopic methods have been implemented to extract stones from the pancreatic duct. Stones that are simple and small can be removed using endoscopic methods like balloon or basket sweeping. Impacted and large stones can be removed by lithotripsy or surgical methods [20]. Numerous studies have shown that most individuals with chronic pancreatitis have symptomatic relief after removing obstruction-causing stones from the primary pancreatic duct [11, 21]. For patients who experience painful obstructive chronic pancreatitis, surgery is considered the most effective method for pain reduction [22]. But, endoscopic methods are preferred over surgical methods because of their less invasive nature, thus considering surgery as the second line of treatment in patients who are not compliant to endoscopic therapy [23]. Other endoscopic options may be regarded as pancreatic sphincterotomy, lithotripsy, etc. The following are the various treatment methods for the removal of pancreatic calculi or stones.

**Figure 2:**
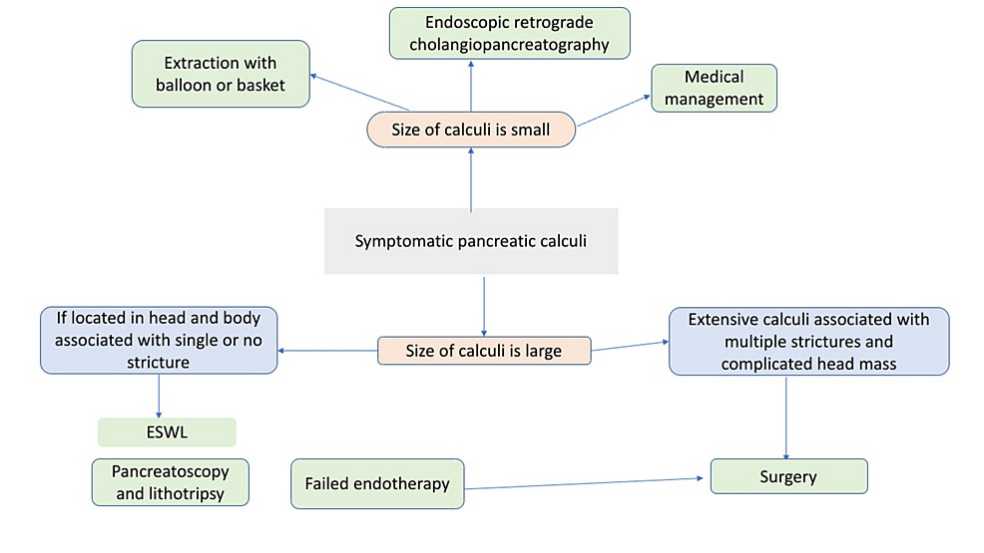
Algorithm for management of pancreatic calculi.

Medical management involves the dissolution of pancreatic stones using chemicals. Endoscopic management involves ERCP, removal of stone, and ESWL. Also, pancreatoscopy, intraductal, and mechanical lithotripsy can sometimes be used. Surgical management includes both drainage and resection methods. The algorithm for various methods of management of pancreatic calculi is shown in Figure 2.

Medical management

Chronic pancreatitis patients, whether or not they have calcifications in the duct, are often managed with oral supplements of pancreatic enzyme, analgesics, and a low-fat diet. These methods are useful in decreasing the production of pancreatic secretions and reducing hydrostatic pressure by preventing the release of cholecystokinin and consequently preventing parenchymal stimulation of the exocrine part [24]. There is no effective medication for dissolving stones. In some cases, trimethadione, an old anticonvulsant, is useful in dissolving pancreatic calcium stones [25]. Since this drug causes hepatotoxicity, it is not used widely. This type of management can be taken into consideration for patients who had poor compliance for all other treatment methods and those who are not compliant with surgical methods [26].

Endoscopy management

The most typical technique to extract small and floating calculi (<5 mm) is ERCP and pancreatic sphincterotomy, accompanied by balloon baskets or trawl. In cases of large and impacted stones (>5 mm), these methods are not useful [27-28]. For painful and large calculi (>5 mm), the first approach should be ESWL and then the removal of fragments of stone at the next ERCP [26]. During ESWL, radiolucent calculi are challenging to target with X-ray. In certain circumstances, the placement of a nasopancreatic tube can help with ESWL stone targeting [29]. The stones situated in the head or body which cause upstream dilation of the main pancreatic duct are preferred for endoscopic removal [30]. Patients who cannot undergo endoscopic excision of pancreatic duct stones include those with significant entire gland stones or those located in side branches without dilation of the main pancreatic duct [31]. The majority of the studies show that pancreatic function is not improved by endoscopic therapy. Still, one study of secretin-enhanced magnetic resonance cholangiopancreatography proposed that endoscopic therapy improves the exocrine function of the pancreas [32].

Pancreatic sphincterotomy

Pancreatic sphincterotomy via the major or minor papilla (with or without a biliary sphincterotomy) to remove pancreatic calculi is performed in the majority of patients. This procedure is required because, in most symptomatic cases, stones in the pancreas have already been unable to pass spontaneously through the intact papilla. A pull-type sphincterotome can be passed over a guide wire, or a needle-knife incision over a pancreatic stent can be used to execute a pancreatic sphincterotomy. The complications can be early and late, including bleeding, perforation, and sphincter stenosis [33, 34]. Additionally, endoscopic balloon dilation was done following pancreatic sphincterotomy to remove sizable radiolucent calculus from the pancreatic duct without stricture, as reported in a case report [35].

Extraction balloons, baskets, and forceps

 The stones from the pancreatic duct are captured and swept with balloons, baskets, and forceps through the small intestinal lumen. The basket is opened to remove the stone from the duct and into the small intestine's lumen. Then mechanical lithotripsy may be used to break up the stone. Extraction balloons are safer than extraction baskets in cases when the downstream duct is smaller than the stone. The risk of trapping can be reduced by deflating the extraction balloon within the duct. Therefore, the usage of an extraction balloon during ERCP is safe and has fewer complications in removing pancreatic duct stones comparatively [36]. Although it is technically challenging to introduce forceps into the pancreatic duct because it can cause its injury, the use of rat tooth forceps that can trap stones 1-2 cm distal to the main duct are somewhat safer as compared to a basket [19].

Dilation and stenting of pancreatic duct strictures

The removal of stone or placement of stent may be facilitated by dilation of stricture. Inflammation, along with fibrosis in the vicinity of the main pancreatic duct, causes benign strictures. A guide wire must be manipulated upstream through the constriction before the use of a balloon or dilating catheter done for stenting or dilatation of the stricture. Chronic pancreatitis causes densely fibrotic pancreatic duct strictures [37].

Intraductal mechanical lithotripsy

When large stones are broken up into small fragments by mechanical lithotripsy, ESWL, or intraductal electrohydraulic lithotripsy (EHL), they can be removed through the papilla with ease. There are more chances of complications in the case of mechanical lithotripsy. Removal of large pancreatic stones has limited success with usage of through-the-scope mechanical lithotripter [38].

Extracorporeal shock wave lithotripsy

This method is preferred for the treatment of large pancreatic calculi [39]. Shock wave energy is the basic principle of ESWL. Shock waves are produced when energy is suddenly released in a confined place. When these waves pass through objects of varied acoustic impedance, compressive stress is generated on the surface of the object. The tensile strength of an object, like calculi, is overcome by the pressure that crumbles the calculi's anterior surface. Some shock waves are reflected when crossed to the posterior surface of the stone and result in further fragmentation of stones [40]. The formation of pancreatic duct calculi in chronic calcific pancreatitis results in upstream hypertension, a rise in the pressure of parenchyma, and ischemia leading to pain. Fragmentation of stones >5 mm in diameter needs to be done for removal from the main duct. In most cases, ESWL was successful for the fragmentation of large calculi, which is then supported by sudden or endoscopic clearance leading to pain relief [9, 39-41]. ESWL is considered safe, non-invasive, and efficient because it causes a reduction in the size of the calculi, and these broken fragments in the pancreatic duct can easily be removed. Indication: the procedure is advised to all patients diagnosed with chronic pancreatitis who has a large pancreatic stone that cannot be removed through endoscopic therapy. The main aim is to reduce the calculi size to <3 mm by breaking them into fragments to remove them with ERCP. The calculi in the head and body of the pancreas are acknowledged first during ESWL. The procedure is not suitable in cases where there are large calculi located in the head, body, and tail of the pancreas or an isolated stone in the tail area, as the risk of collateral damage is high in the spleen. Protocol: a lithotripter of the third generation is generally used to perform ESWL, which generates shock waves by electromagnetic shock waves. A maximum of 5,000-6,000 shocks with a 15-16 kV intensity, given at a frequency of 90 shocks per minute, are allowed every session. The process is repeated every day until the desired fragmentation is obtained [40]. ESWL was successfully performed with parenteral sedation, complete IV analgesia, and general anesthesia. Recently, some facilities have successfully used a portable or mobile small lithotripter for ESWL [42]. Limitations: To establish the role of ESWL in the removal of calculi from the main duct in chronic calculi pancreatitis patients, long-term follow-up is required [43]. In some cases, there may be incomplete fragmentation of calculi. In such cases, the patients should be directed to surgery. Also, there may be a recurrence of the condition after performing ESWL [23, 44]. It would be very helpful to utilize pharmacological drugs that can stop this reformation to minimize stone recurrence and lessen the necessity for additional interventions. In conclusion, ESWL is an effective method for the removal of large calculi from the pancreatic duct of patients who have chronic calculi pancreatitis and provides effective pain relief. In addition to preventing the necessity for surgery, it is possible that ESWL is performed at a young age which is then continued by intense medical therapy that will change the path of patients with chronic calculi pancreatitis [9].

Electrohydraulic lithotripsy

This procedure can also be done for the fragmentation of pancreatic calculi. It includes a bipolar probe and a charge generator which produces sparks. A vapor plasma, followed by a cavitation bubble that oscillates around the tip of the probe, is created. Three different shock waves are produced together. Due to the rapid growth of vapor plasma, the first wave is generated, while due to the rebound of the cavitation bubble second and third wave is generated. Nearby stones absorb the energy of these high-frequency hydraulic pressure waves, which causes the stones to break [45].

Laser lithotripsy

In this procedure, the surface of the stone is focused by laser light at a fixed wavelength to produce wave-mediated fragmentation. Due to its high costs, reduced portability, and availability of other feasible methods, the use of laser technology is limited. Stone material melts and is ejected from the stone's surface. A vapor bubble is then produced by the water absorption of laser energy which removes the stone material [46].

Surgical management

The pancreatic duct calculi can be eliminated by surgical management methods. The aim of simpler methods includes the removal of calculi obstructing the duct, obstructed ducts decompression, and preserving tissue of the pancreas as well as nearby organs. The selection of surgical management of pancreatic calculi depends on a lot of factors like the pancreatic duct diameter, presence of strictures in the main duct, associated pseudocyst, related cancer concerns, the extent of obstruction by the stones, and tolerance of the operation. Surgical treatment is considered the second line of management in patients who are not compliant to endoscopic therapy [19].

## Conclusions

Pancreatic calculi is the natural sequel of the progressing condition of chronic pancreatitis. Pain being the most common clinical feature of chronic pancreatitis is relieved by various methods like surgical and endoscopic for stone removal. Small size stones are extracted by pancreatic sphincterotomy, followed by extraction balloons and baskets. Large-size stones need fragmentation which is done by ESWL and other intraductal lithotripsy methods. This type of management is considered the first line of treatment and is considered effective for pain relief. In cases that fail to respond to endoscopic treatment, surgery is considered the second line of management.

## References

[1] Nabi Z, Lakhtakia S (2021). Endoscopic management of chronic pancreatitis. Dig Endosc.

[2] Oza VM, Kahaleh M (2013). Endoscopic management of chronic pancreatitis. World J Gastrointest Endosc.

[3] Kwek AB, Ang TL, Maydeo A (2014). Current status of endotherapy for chronic pancreatitis. Singapore Med J.

[4] Kamat R, Gupta P, Rana S (2022). Imaging in chronic pancreatitis: state of the art review. Indian J Radiol Imaging.

[5] Tandan M, Talukdar R, Reddy DN (2016). Management of pancreatic calculi: an update. Gut Liver.

[6] Kleeff J, Whitcomb DC, Shimosegawa T (2017). Chronic pancreatitis. Nat Rev Dis Primers.

[7] Khalid A, Whitcomb DC (2002). Conservative treatment of chronic pancreatitis. Eur J Gastroenterol Hepatol.

[8] Ammann RW, Muench R, Otto R (1988). Evolution and regression of pancreatic calcification in chronic pancreatitis: a prospective long-term study of 107 patients. Gastroenterology.

[9] Ong WC, Tandan M, Reddy V, Rao GV, Reddy N (2006). Multiple main pancreatic duct stones in tropical pancreatitis: safe clearance with extracorporeal shockwave lithotripsy. J Gastroenterol Hepatol.

[10] Steer ML, Waxman I, Freedman S (1995). Chronic pancreatitis. N Engl J Med.

[11] Sherman S, Lehman GA, Hawes RH (1991). Pancreatic ductal stones: frequency of successful endoscopic removal and improvement in symptoms. Gastrointest Endosc.

[12] Jin CX, Naruse S, Kitagawa M (2002). Pancreatic stone protein of pancreatic calculi in chronic calcified pancreatitis in man. JOP.

[13] Pitchumoni CS, Viswanathan KV, Gee Varghese PJ, Banks PA (1987). Ultrastructure and elemental composition of human pancreatic calculi. Pancreas.

[14] Tanaka T, Miura Y, Ichiba Y, Itoh H, Dohi K (1992). Experimental pancreatolithiasis: association with chronic alcoholic pancreatitis. Am J Gastroenterol.

[15] Midha S, Khajuria R, Shastri S, Kabra M, Garg PK (2010). Idiopathic chronic pancreatitis in India: phenotypic characterisation and strong genetic susceptibility due to SPINK1 and CFTR gene mutations. Gut.

[16] Feldman M, Friedman LS, Brandt LJ (2020). Sleisenger and Fordtran’s Gastrointestinal and Liver Disease Pathophysiology, Diagnosis, Management.

[17] Luetmer PH, Stephens DH, Ward EM (1989). Chronic pancreatitis: reassessment with current CT. Radiology.

[18] Kahl S, Glasbrenner B, Leodolter A, Pross M, Schulz HU, Malfertheiner P (2002). EUS in the diagnosis of early chronic pancreatitis: a prospective follow-up study. Gastrointest Endosc.

[19] Choi EK, Lehman GA (2012). Update on endoscopic management of main pancreatic duct stones in chronic calcific pancreatitis. Kor J Intern Med.

[20] Farnbacher MJ, Schoen C, Rabenstein T (2002). Pancreatic duct stones in chronic pancreatitis: criteria for treatment intensity and success. Gastrointest Endosc.

[21] Dumonceau JM, Devière J, Le Moine O (1996). Endoscopic pancreatic drainage in chronic pancreatitis associated with ductal stones: long-term results. Gastrointest Endosc.

[22] Cahen DL, Gouma DJ, Nio Y (2007). Endoscopic versus surgical drainage of the pancreatic duct in chronic pancreatitis. N Engl J Med.

[23] Delhaye M, Arvanitakis M, Bali M, Matos C, Devière J (2005). Endoscopic therapy for chronic pancreatitis. Scand J Surg.

[24] Isaksson G, Ihse I (1983). Pain reduction by an oral pancreatic enzyme preparation in chronic pancreatitis. Dig Dis Sci.

[25] Noda A, Okuyama M, Murayama H, Takeuchi K, Yokota T, Kobayashi T, Takayama T (1994). Dissolution of pancreatic stones by oral trimethadione in patients with chronic calcific pancreatitis. J Gastroenterol Hepatol.

[26] Dumonceau JM, Delhaye M, Tringali A (2012). Endoscopic treatment of chronic pancreatitis: European Society of Gastrointestinal Endoscopy (ESGE) Clinical Guideline. Endoscopy.

[27] Tandan M, Reddy DN, Santosh D (2010). Extracorporeal shock wave lithotripsy and endotherapy for pancreatic calculi-a large single center experience. Indian J Gastroenterol.

[28] Lehman GA (2002). Role of ERCP and other endoscopic modalities in chronic pancreatitis. Gastrointest Endosc.

[29] Tandan M, Nageshwar Reddy D (2013). Endotherapy in chronic pancreatitis. World J Gastroenterol.

[30] Smits ME, Rauws EA, Tytgat GN (1996). Endoscopic treatment of pancreatic stones in patients with chronic pancreatitis. Gastrointest Endosc.

[31] Tringali A, Boskoski I, Costamagna G (2008). The role of endoscopy in the therapy of chronic pancreatitis. Best Pract Res Clin Gastroenterol.

[32] Bali MA, Sztantics A, Metens T, Arvanitakis M, Delhaye M, Devière J, Matos C (2005). Quantification of pancreatic exocrine function with secretin-enhanced magnetic resonance cholangiopancreatography: normal values and short-term effects of pancreatic duct drainage procedures in chronic pancreatitis. Initial results. Eur Radiol.

[33] Cotton PB, Lehman G, Vennes J (1991). Endoscopic sphincterotomy complications and their management: an attempt at consensus. Gastrointest Endosc.

[34] Masci E, Toti G, Mariani A (2001). Complications of diagnostic and therapeutic ERCP: a prospective multicenter study. Am J Gastroenterol.

[35] Maydeo A, Bhandari S, Bapat M (2009). Endoscopic balloon sphincteroplasty for extraction of large radiolucent pancreatic duct stones (with videos). Gastrointest Endosc.

[36] Adler DG, Conway JD, Farraye FA (2009). Biliary and pancreatic stone extraction devices. Gastrointest Endosc.

[37] Eisendrath P, Devière J (1999). Expandable metal stents for benign pancreatic duct obstruction. Gastrointest Endosc Clin N Am.

[38] Freeman ML (1996). Mechanical lithotripsy of pancreatic duct stones. Gastrointest Endosc.

[39] Delhaye M, Vandermeeren A, Baize M (1992). Extracorporeal shock-wave lithotripsy of pancreatic calculi. Gastroenterology.

[40] Tandan M, Reddy DN (2011). Extracorporeal shock wave lithotripsy for pancreatic and large common bile duct stones. World J Gastroenterol.

[41] Guda NM, Partington S, Freeman ML (2005). Extracorporeal shock wave lithotripsy in the management of chronic calcific pancreatitis: a meta-analysis. JOP.

[42] Milovic V, Wehrmann T, Dietrich CF, Bailey AA, Caspary WF, Braden B (2011). Extracorporeal shock wave lithotripsy with a transportable mini-lithotripter and subsequent endoscopic treatment improves clinical outcome in obstructive calcific chronic pancreatitis. Gastrointest Endosc.

[43] Adamek HE, Jakobs R, Buttmann A, Adamek MU, Schneider AR, Riemann JF (1999). Long term follow up of patients with chronic pancreatitis and pancreatic stones treated with extracorporeal shock wave lithotripsy. Gut.

[44] Inui K, Tazuma S, Yamaguchi T (2005). Treatment of pancreatic stones with extracorporeal shock wave lithotripsy: results of a multicenter survey. Pancreas.

[45] Koch H, Stolte M, Walz V (1977). Endoscopic lithotripsy in the common bile duct. Endoscopy.

[46] Maydeo A, Kwek BE, Bhandari S, Bapat M, Dhir V (2011). Single-operator cholangioscopy-guided laser lithotripsy in patients with difficult biliary and pancreatic ductal stones (with videos). Gastrointest Endosc.

